# Association between birth weight and objectively measured sedentary time is mediated by central adiposity: data in 10,793 youth from the International Children’s Accelerometry Database^1^,^2^,^3^

**DOI:** 10.3945/ajcn.114.103648

**Published:** 2015-04-01

**Authors:** Maria Hildebrand, Elin Kolle, Bjørge H Hansen, Paul J Collings, Katrien Wijndaele, Katarzyna Kordas, Ashley R Cooper, Lauren B Sherar, Lars Bo Andersen, Luis B Sardinha, Susi Kriemler, Pedro Hallal, Esther van Sluijs, Ulf Ekelund

**Affiliations:** 1 From the Department of Sports Medicine, Norwegian School of Sport Sciences, Oslo, Norway (MH, EK, BHH, LBA, and UE); the Medical Research Council Epidemiology Unit, University of Cambridge, Cambridge, United Kingdom (PJC, KW, EvS, and UE); the School of Social and Community Medicine (KK) and the Centre for Exercise, Nutrition & Health Sciences, School for Policy Studies (ARC), University of Bristol, Bristol, United Kingdom; the School of Sport, Exercise and Health Sciences, Loughborough University, Loughborough, United Kingdom (LB Sherar); the Institute of Sport Science and Clinical Biomechanics, University of Southern Denmark, Odense, Denmark (LBA); the Department of Sport and Health, University of Lisbon, Lisbon, Portugal (LB Sardinha); the Institute of Social and Preventive Medicine, University of Zürich, Zürich, Switzerland (SK); and the Department of Physical Education, Federal University of Pelotas, Pelotas, Brazil (PH).

**Keywords:** abdominal adiposity, birth weight, sedentary time, youth, mediation, accelerometry

## Abstract

**Background:** Birth weight is an early correlate of disease later in life, and animal studies suggest that low birth weight is associated with reduced activity and increased sedentary time. Whether birth weight predicts later sedentary time in humans is uncertain.

**Objectives:** We examined the relation between birth weight and sedentary time in youth and examined whether this association was mediated by central adiposity.

**Design:** We used pooled cross-sectional data from 8 observational studies conducted between 1997 and 2007 that consisted of 10,793 youth (boys: 47%) aged 6–18 y from the International Children’s Accelerometry Database. Birth weight was measured in hospitals or maternally reported, sedentary time was assessed by using accelerometry (<100 counts/min), and abdominal adiposity (waist circumference) was measured according to WHO procedures. A mediation analysis with bootstrapping was used to analyze data.

**Results:** The mean (±SD) time spent sedentary was 370 ± 91 min/d. Birth weight was positively associated with sedentary time (*B* = 4.04, *P* = 0.006) and waist circumference (*B* = 1.59, *P* < 0.001), whereas waist circumference was positively associated with sedentary time (*B* = 0.82, *P* < 0.001). Results of the mediation analysis showed a significant indirect effect of birth weight on sedentary time through waist circumference (*B*: 1.30; 95% bias-corrected CI: 0.94, 1.72), and when waist circumference was controlled for, the effect of birth weight on sedentary time was attenuated by 32% (*B* = 2.74, *P* = 0.06).

**Conclusion:** The association between birth weight and sedentary time appears partially mediated by central adiposity, suggesting that both birth weight and abdominal adiposity may be correlates of sedentary time in youth.

## INTRODUCTION

The Developmental Origins of Health and Disease hypothesis suggests that nonoptimal growth and environmental conditions during fetal life may result in permanent changes in the body’s structure, function, and metabolism (1). These irreversible adaptations can increase risk of diseases later in life, and birth weight, which is used as an indicator of intrauterine growth and the prenatal environment (2), is inversely associated with increased risk of all-cause mortality (3), cardiovascular disease (4), and type 2 diabetes later in life (5). In addition, a low birth weight is associated with reduced muscle mass and strength (6, 7) as well as lower aerobic fitness later in life (7–9). A lower probability of undertaking leisure-time physical activity later in life in individuals born with low or high birth weight was also suggested (10).

Animal studies showed that the offspring of undernourished mothers are less active and more sedentary than offspring born within normal birth weights (11, 12). In humans, the current knowledge on whether birth weight is associated with behaviors such as sedentary time is limited. One study that used an objective measure of sedentary time showed no associations with birth weight; however, analyses were limited to subjects born in the low-to-normal weight spectrum of birth weights (13). In addition, knowledge about the mechanisms that may underpin a potential association between birth weight and sedentary time is scant; in the current study, we hypothesized that central adiposity is one such mechanism.

A higher birth weight is associated with increased risk of obesity (14), greater overall fat mass (15), and higher BMI (16), whereas a lower birth weight is related to a higher percentage of body fat (17) and central adipose tissue in youth (16, 18, 19). Therefore, it was suggested that both overnutrition and undernutrition during fetal life can trigger pathways responsible for obesity later in life (19). In addition, obesity appears to be associated with and shares the same pathophysiologic mechanisms as low cardiorespiratory fitness and muscle mass (20), and although studies that used both objective (21–24) and subjective (25) measures suggested that higher amounts of sedentary time may not predict central adiposity, the reverse was reported in young people (21, 23, 24). Therefore, it is plausible that central adiposity may mediate a potential association between birth weight and sedentary time in youth.

Because of the high amount of time spent sedentary in youth (26, 27) and the potential independent harmful effects of excessive sedentary behavior on numerous health outcomes later in life (25, 28, 29), an understanding of potential correlates and determinants of this behavior is important to provide evidence for public health interventions aimed at reducing sedentary time. Thus, the aims of this study were to examine the relation between birth weight and objectively measured sedentary time and whether this association is mediated by central adiposity.

## METHODS

### Study design and participants

The International Children’s Accelerometry Database (ICAD)^4^ (http://www.mrc-epid.cam.ac.uk/Research/Studies/) was established to pool data on objectively measured physical activity and sedentary time from observational studies in youth worldwide. Aims, design, inclusion criteria, and methods of the ICAD project have been described in detail previously (30). Briefly, the ICAD consists of re-analyzed and pooled accelerometer data combined with phenotypic information in ∼32,000 young people aged 3–18 y. Ethical approval was granted for each individual study, and all participants have provided informed parental consent. Formal data-sharing agreements were established, and all partners consulted with their individual research boards to confirm sufficient ethical approval had been attained for contributing data. For this study, data from 8 studies conducted between 1997 and 2007, in which measured or maternally reported birth weight, measured waist circumference, and sedentary time were available (*n* = 10,793) were included (31–40). This subsample (aged 6–18 y) differed slightly from the whole ICAD sample in terms of time spent sedentary (+16 min/d; 4.3%; *P* < 0.001) and waist circumference (+1 cm; 1.5%; *P* < 0.001).

### Measurements

A detailed description of the assessment of sedentary time and physical activity is available elsewhere (30). Accelerometer data in the ICAD were re-analyzed centrally in a standardized manner with specialist software (KineSoft Software, version 3.3.20; Kinesoft.org) (30) and processed in 60-s epochs to provide comparable physical activity outcomes across studies.

The Pelotas study used a 24-h wear protocol (34, 35), whereas the other studies asked participants to wear the accelerometer during waking hours only. To avoid accelerometer data being influenced by the increased wear time, accelerometry data were excluded for the overnight period between 2400 and 0700 in the Pelotas study. Children with ≥3 d with 600 min of measured monitor wear time between 0700 and midnight were included. Nonwear time was defined as 60 min of consecutive zeroes, with the allowance for 2 min of nonzero interruptions, terminated at the third nonzero interruption (41, 42). Overall physical activity was calculated as total counts over the wear period and expressed in counts per minute. The time spent sedentary was defined as all minutes with <100 counts/min (43), whereas the time spent in moderate-to-vigorous physical activity (MVPA) was defined as minutes with >3000 counts/min (44). Both sedentary time and time spent in MVPA are expressed in minutes per day.

Height and weight were measured by using a standardized procedure across studies. BMI (weight divided by height squared) was calculated for each participant, and age- and sex specific BMI cutoffs were used to categorize participants as normal weight, overweight, or obese (45). Waist circumference was used as a surrogate measure for abdominal adiposity and measured according to WHO procedures by using a metal anthropometric tape midway between the lower rib margin an iliac crest at the end of a gentle expiration (46). Birth weight was directly measured (Pelotas study) or maternally reported, which has been shown to be highly correlated with measured birth weight (47).

### Statistical analyses

Means (±SDs) are shown for descriptive variables. An independent *t* test was used to compare descriptive data between sexes.

We used resampling strategies and the macro presented by Preacher and Hayes (48) to assess whether waist circumference (cm) acts as a potential mediator of the association between birth weight (kg) and sedentary time (min/d). Bootstrapping is a nonparametric resampling procedure that does not require the assumption of normality of the sampling distribution and is a recommended method of obtaining confidence limits for indirect effects. The method involves repeated sampling from the data set, and the indirect effect is estimated in each resampled data set (48). In the unstandardized regression equation (ordinary least-squares regression), birth weight was modeled as the predictor, sedentary time was modeled as the outcome, waist circumference was modeled as the mediator, and sex, age, study, and monitor wear time were modeled as covariates. Analyses were used to determine the total (*c* path) and direct effect (*c*′ path) of birth weight on sedentary time and estimate the mediating role of waist circumference (the *a* × *b* products; indirect effect of the independent variable on the dependent variable through the mediator). In the current study, a 95% bias-corrected CI (bCI) for each *a* × *b* product was obtained with 5000 bootstrap resamples and used to assess whether waist circumference mediated the association between birth weight and sedentary time. A significant indirect effect via the mediator between birth weight and sedentary time was determined if the 95% bCI did not overlap zero.

We did not have data on gestational age, and therefore, we could not differentiate between participants with low birth weight because of premature birth or growth restriction. The sample consisted of 553 participants who could be considered premature (birth weight <2.5 kg), and therefore, we performed sensitivity analyses by excluding participants with birth weight <2.5 kg.

We examined whether the association between birth weight and sedentary time was modified by sex or age by including the interaction term birth weight × sex and birth weight × age; however, no significant interactions were observed (*P* > 0.10). The association between different categories of birth weights and sedentary time is displayed graphically for illustrative purposes and presented as means and 95% CIs of the sedentary time for each birth weight group (see Results section). Birth weight was divided into 6 categories as follows: <2.75 kg (*n* = 1164), 2.75–3.25 kg (*n* = 2822), 3.26–3.75 kg (*n* = 4160), 3.76–4.25 kg (*n* = 2117), 4.26–4.75 kg (*n* = 449), and >4.75 kg (*n* = 81), and the birth-weight category 3.26–3.75 kg was chosen as the reference category because it contained the largest proportion of participants and in agreement with previous studies (10). All analyses were performed with SPSS v 18.0 software (SPSS).

## RESULTS

Descriptive statistics by study and sex are summarized in **Tables 1** and **2**, respectively. Overall, 79.3% of children were categorized as normal weight, 15.9% of children were categorized as overweight, and 4.8% of children were categorized as obese. The mean birth weight differed by 0.33 kg between studies, and the lowest mean (±SD) birth weight (3.22 ± 0.54 kg) was observed from the cohort who represented a low- and middle-income country (Brazil). Children’s sedentary time and physical activity were monitored for an average of 5.3 ± 1.3 d. Overall, the average time spent sedentary was 370 ± 91 min/d, whereas, on average, 56 ± 30 min/d were spent in MVPA. Boys spent, on average, significantly more time in MVPA than did girls (66 compared with 46 min/d, respectively; *P* < 0.001) and less time sedentary than did girls (360 compared with 380 min/d, respectively; *P* < 0.001).

**TABLE 1:** Descriptive statistics of the 8 included studies (*n* = 10,793)^1^

Study, country (reference)	Year	*n* (% boys)	Age,^2^ y	Height, cm	Weight, kg	BMI,^3^ kg/m^2^	Birth weight, kg	Total physical activity, counts/min	Sedentary time,^4^ min/d	MVPA,^5^ min/d
ALSPAC, United Kingdom (32)	2003–2004	5808 (48)	11–15	151.8 ± 8.1^6^	44.4 ± 10.5	19.1 ± 3.4	3.41 ± 0.55	588 ± 191	371 ± 75	57 ± 28
EYHS, Denmark (31, 33)	1997–1998	1162 (45)	8–18	148.5 ± 15.3	41.8 ± 14.3	18.4 ± 3.1	3.40 ± 0.59	562 ± 253	384 ± 125	50 ± 33
EYHS, Estonia (31)	1998–1999	557 (44)	8–17	151.8 ± 17.1	44.0 ± 15.6	18.4 ± 3.1	3.55 ± 0.59	631 ± 251	352 ± 111	63 ± 38
EYHS, Norway (31, 40)	1999–2000	350 (51)	9–10	139.3 ± 6.3	33.3 ± 5.9	17.1 ± 2.3	3.46 ± 0.59	709 ± 305	339 ± 108	69 ± 37
EYHS, Portugal (31, 36)	1999–2000	547 (51)	9–18	147.1 ± 14.6	43.3 ± 14.4	19.5 ± 3.7	3.39 ± 0.52	553 ± 233	390 ± 109	52 ± 35
KISS, Switzerland (39)	2005; 2006	307 (46)	6–13	136.4 ± 13.0	33.0 ± 10.1	17.3 ± 2.8	3.36 ± 0.57	576 ± 212	307 ± 112	74 ± 30
Pelotas, Brazil (34, 35)	2006–2007	426 (53)	13–14	157.9 ± 8.4	50.9 ± 12.1	20.3 ± 3.8	3.22 ± 0.54	320 ± 118	389 ± 132	40 ± 26
SPEEDY, United Kingdom (37, 38)	2007	1636 (44)	10–11	141.1 ± 6.7	36.5 ± 8.3	18.2 ± 3.1	3.35 ± 0.58	594 ± 190	371 ± 69	50 ± 24

1 ALSPAC, Avon Longitudinal Study of Parents and Children; EYHS, European Youth Heart Study; KISS, Kinder Sportstudie; MVPA, moderate-to-vigorous physical activity; SPEEDY, Sport, Physical Activity and Eating Behavior: Environmental Determinants in Young People.

2 All values are ranges.

3 BMI is calculated as weight divided by height squared.

4 The cutoff for sedentary time was <100 counts/min.

5 The cutoff for MVPA was >3000 counts/min.

6 Mean ± SD (all such values).

**TABLE 2:** Baseline descriptive statistics of the sample stratified by sex (*n* = 10,793)

	Boys (*n* = 5092)	Girls (*n* = 5701)	*P*^1^
Age, y	11.5 ± 1.6^2^	11.5 ± 1.7	0.63
Weight, kg	42.0 ± 12.1	42.8 ± 11.6	0.001
Height, cm	149.1 ± 11.9	148.8 ± 10.7	0.13
Waist circumference, cm	66.7 ± 9.2	65.7 ± 9.2	<0.001
BMI^3^^,^^4^	18.6 ± 3.2	19.0 ± 3.4	<0.001
Normal weight,^3^ *n* (%)	4109 (80.8)	4432 (77.9)	—
Overweight,^3^ *n* (%)	767 (15.1)	946 (16.6)	—
Obese,^3^ *n* (%)	207 (4.1)	312 (5.5)	—
Birth weight, g	3459 ± 584	3345 ± 535	<0.001
Total physical activity, counts/min	637 ± 231	528 ± 186	<0.001
Sedentary time,^5^ min/d	360 ± 91	380 ± 90	<0.001
MVPA,^6^ min/d	66 ± 33	46 ± 23	<0.001
Wear time, d	5.4 ± 1.4	5.3 ± 1.3	0.96

1 *P* values denote differences between sex and were determined by using a *t* test for normally distributed continuous variables.

2 Mean ± SD (all such values).

3 *n* = 5083 and 5690 for boys and girls, respectively.

4 Age- and sex-specific BMI cutoffs proposed by Cole et al. (45) were used.

5 The cutoff for sedentary time was <100 counts/min.

6 MVPA, moderate-to-vigorous physical activity. The cutoff for MVPA was >3000 counts/min.

**FIGURE 1:**
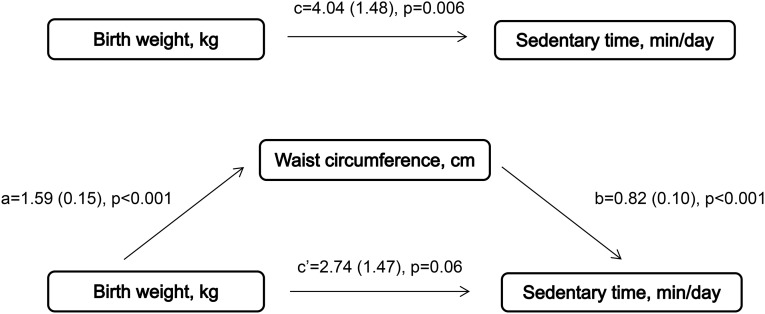
Unstandardized regression coefficients (±SEs) in regression analyses included in the mediator model between birth weight, waist circumference, and sedentary time (*n* = 10,793). Analyses were performed by using ordinary least-squares regression and adjusted for sex, age, study, and monitor wear time. The paths represent the difference in waist circumference (cm) per 1-kg increase in birth weight (path a), difference in sedentary time (min/d) per 1-cm increase in waist circumference (path b), and differences in sedentary time (min/d) per 1-kg increase in birth weight with (path c′) and without (path c) adjustment for waist circumference.

**FIGURE 2:**
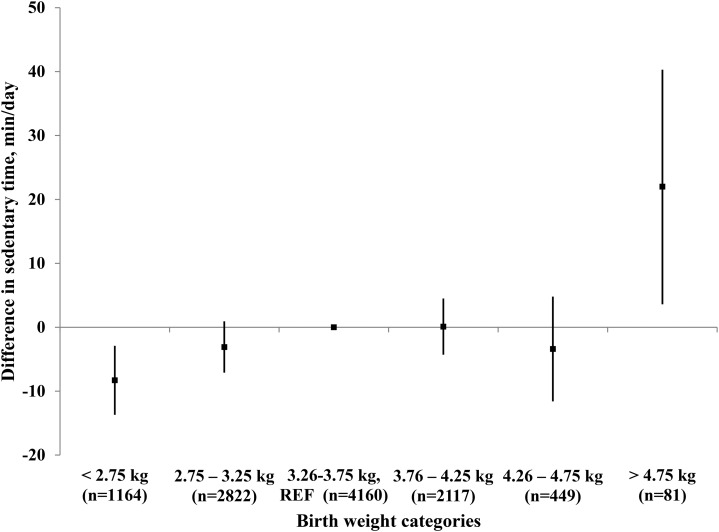
Mean (95% CI) differences expressed in sedentary min/d stratified by birth-weight categories compared with the reference group (birth weight: 3.26–3.75 kg; *n* = 10,793; *P*-trend = 0.003) adjusted for sex, age, study, and monitor wear time (ordinary least-squares regression). REF, reference.

**Figure 1** shows the separate regression analyses conducted to assess each component of the proposed mediation model among variables. Birth weight was associated with sedentary time, and a 1-kg increase in birth weight was associated with 4 more minutes spent sedentary per day (*c* path; *B* = 4.04, *P* = 0.006). When this association was modeled graphically, the association seemed to be mainly driven by individuals in the extreme categories of birth weight (<2.75 and >4.75 kg) (**Figure 2**). In addition, birth weight was positively associated with waist circumference (*a* path; *B* = 1.59, *P* < 0.001), and waist circumference was positively associated with sedentary time (*b* path; *B* = 0.82, *P* < 0.001). Results of the mediation analysis confirmed the mediating role of waist circumference in the association between birth weight and sedentary time (*a* × *b* path; *B*: 1.30; 95% bCI: 0.94, 1.72). Furthermore, our results showed that the direct effect of birth weight on sedentary time was attenuated by 32% (*c*′ path; *B* = 2.74, *P* = 0.06) when controlling for waist circumference, which suggested partial mediation.

In sensitivity analyses, with the exclusion of individuals with birth weight <2.5 kg, results were mainly unchanged. Birth weight was associated with sedentary time (*c* path; *B* = 4.66, *P* = 0.01) and waist circumference (*a* path; *B* = 2.15, *P* < 0.001), and waist circumference was positively associated with sedentary time (*b* path; *B* = 0.81, *P* < 0.001). In addition, results of the mediation analysis confirmed the mediating role of waist circumference in the association between birth weight and sedentary time (*a* × *b* path; *B*: 1.74; 95% bCI: 1.26, 2.30) and supported partial mediation (*c*′ path; *B* = 2.92, *P* = 0.11).

## DISCUSSION

This study examined whether birth weight acts as a correlate of sedentary time in youth and whether this association is mediated by waist circumference. The results suggested that the association between birth weight and sedentary time was partially mediated by waist circumference in young people aged 6–18 y.

We proposed that central adiposity could be an underlying mechanism of the association between birth weight and sedentary time for several reasons. Birth weight appears to be associated with several measures of adiposity (15, 17, 19), muscle mass (6), and aerobic fitness later in life (9), which are factors that are potentially related to sedentary time. In addition, obesity has been related to aerobic fitness, muscle mass (20), and objectively measured sedentary time in youth (21). Our participants covered a wide range of the birth-weight spectrum (645–5750 g), and even though we showed significant differences in weight and birth weight between sexes, differences were small (<0.8 kg or 115 g) and may not have been clinically important. The results showed that the association was partially mediated by waist circumference, and the effect of birth weight on sedentary time was reduced by 32% when controlling for the suggested mediator. In addition, controlling for waist circumference made the association between birth weight and sedentary time nonsignificant. This result could have been due to multicollinearity between birth weight and waist circumference, and with a larger sample size, this result could have reached significance. However, when the significance was disregarded, the magnitude of the association was small, and a 1-kg increase in birth weight was associated with only 2.7 more minutes spent sedentary per day. This observation was opposite to some previous observations in animal models (11, 12) but consistent with the results of a previous study in subjects born with low to normal birth weights; these results were also unable to show an association between birth weight and sedentary time (13). Although sedentary behavior is different from not performing enough physical activity (49), studies that used objectively measured physical activity were also unable to show an association between birth weight and later levels of physical activity in youth (13, 50–53), and it has been suggested that, across the normal birth weight spectrum, physical activity and sedentary time in youth are more influenced by environmental and behavioral factors than birth weight (50). In contrast, the magnitude of the association between birth weight and sedentary time (and physical activity) could have been underestimated because of the inherent variability of youth’s sedentary time and physical activity. A few days of measurement may not have been representative of the true durations of sedentary time in our participants (54, 55). It is also unknown whether the magnitude of the association between birth weight and sedentary time changes by age and may become apparent in adulthood.

A limitation regarding the mediating role of abdominal adiposity in the current study was that waist circumference was measured at the same point in time as sedentary time. Thus, the temporal sequence between waist circumference and sedentary time was not possible to establish. However, it was previously shown that higher waist circumference predicted higher amounts of sedentary time in youth (21, 23), whereas sedentary time did not predict adiposity (21–23, 56–58). In addition, a re-analysis of the data modeling sedentary time as the mediator and waist circumference as the outcome showed that, although significant, sedentary time attenuated the effect of birth weight on waist circumference by only 2% (compared with 32% when waist circumference was the mediator; data not shown), which supported the use of waist circumference as a mediator in our analyses. Nevertheless, our results did not dismiss the possibility of a reverse causation (i.e., a bidirectional association between sedentary time and abdominal adiposity).

Previous studies showed that lower birth weight is associated with central adipose tissue in childhood (16, 18, 19). In the current study, higher birth weight was associated with higher waist circumference; however, this association was attenuated by current BMI (results not shown). The interpretation of associations between birth weight and obesity later in life, which were substantially attenuated after adjustment for current body size (e.g., BMI), suggested that postnatal growth and the change in size (e.g., weight percentile crossing) between time points may be more-important factors on the causal pathway leading to abdominal adiposity than birth weight per se (59). As a result, public health strategies intended to influence the biology of fetal growth are most likely not the most essential approaches, maybe with the exception for obese pregnant women and those who have gestational diabetes, because both obesity during pregnancy and gestational diabetes are associated with large-for-gestational-age infants (60, 61). Rather, strategies that aim to affect other factors such as postnatal weight gain are likely to be more successful to moderate risk of obesity and metabolic diseases later in life because rapid infant weight is associated with childhood obesity (62).

There were several strengths of this study, including objectively measured sedentary time, a wide range of birth weights, and a large and diverse sample representing different geographical and cultural locations. Even though accelerometer data were re-analyzed in a standardized manner, and all analyses were adjusted for wear time, it was possible that differences in accelerometer wear protocol influenced the results. One of the included studies used a 24-h monitor wear protocol (34, 35), and even if this difference was accounted for by excluding time between 2400 and 0700, this protocol may have influenced the amount of time defined as sedentary time in this specific study. However, when the data were re-analyzed after the exclusion of this study, findings were largely unchanged (data not shown).

There were some limitations that warrant consideration in interpreting the results of the current study. The accelerometer is regarded as a valid tool for measuring physical activity and sedentary time (43, 63); however, a hip-placed monitor can be less effective in distinguishing sedentary postures, such as lying and sitting, from other light-intensity activities performed while standing and do not accurately capture upper-body movement, cycling (64), or other activities when the monitor is removed (e.g., water-based activities). Finally, nonwear time was subtracted from wear time and, consequently, prolonged quiet sitting could potentially have been considered nonwear time, thereby leading to an underestimation of sedentary time. The amount of time spent sedentary may have differed between weekdays and weekend days. However, >85% of participants in our data set had ≥1 d of valid accelerometer data during a weekend day, and therefore, we believe it was unlikely that this difference affected our analyses of associations between birth weight and sedentary time. With the exception of one study, birth weight was reported retrospectively. However, it has been suggested that maternally recalled birth weight is highly correlated with measured birth weight and is sufficiently accurate to use in epidemiologic studies (47). Waist circumference was used as the outcome for abdominal adiposity. Despite abdominal adiposity being recognized as an important determinant for disease and mortality (65), a more-detailed measure of body composition may be preferred. Finally, we could not exclude that other unmeasured confounding variables including genotype, infant rapid weight gain, socioeconomic status, and mothers’ BMI might explain our findings. Future prospective studies with several measures of the mentioned confounder variables are needed to examine the potential mediating or modifying effects on the relation between birth weight and later sedentary time at different ages.

In conclusion, the prevalence of sedentary time in youth is of public health concern, and therefore, it is important to understand potential biological and behavioral correlates of this behavior. The results suggest that birth weight is positively associated with sedentary time; however, the association appears partially mediated by central adiposity. Therefore, the targeting of both birth weight and obesity may be an important public health strategy to prevent excessive sedentary time in youth.

## Footnotes

4 Abbreviations used: bCI, bias-corrected CI; ICAD, International Children’s Accelerometry Database; MVPA, moderate-to-vigorous physical activity.

## References

[1] Wadhwa PD, Buss C, Entringer S, Swanson JM Developmental origins of health and disease: brief history of the approach and current focus on epigenetic mechanisms. Semin Reprod Med 2009;27:358–68. 19711246 10.1055/s-0029-1237424 PMC2862635

[2] Baker JL, Olsen LW, Sorensen TI Weight at birth and all-cause mortality in adulthood. Epidemiology 2008;19:197–203. 18300695 10.1097/EDE.0b013e31816339c6

[3] Risnes KR, Vatten LJ, Baker JL, Jameson K, Sovio U, Kajantie E, Osler M, Morley R, Jokela M, Painter RC, Birthweight and mortality in adulthood: a systematic review and meta-analysis. Int J Epidemiol 2011;40:647–61. 21324938 10.1093/ije/dyq267

[4] Zhang Z, Kris-Etherton PM, Hartman TJ Birth weight and risk factors for cardiovascular disease and type 2 diabetes in US children and adolescents: 10 year results from NHANES. Matern Child Health J 2014;18:1423–32. 24241968 10.1007/s10995-013-1382-y

[5] Whincup PH, Kaye SJ, Owen CG, Huxley R, Cook DG, Anazawa S, Barrett-Connor E, Bhargava SK, Birgisdottir BE, Carlsson S, Birth weight and risk of type 2 diabetes: a systematic review. JAMA 2008;300:2886–97. 19109117 10.1001/jama.2008.886

[6] Dodds R, Denison HJ, Ntani G, Cooper R, Cooper C, Sayer AA, Baird J Birth weight and muscle strength: a systematic review and meta-analysis. J Nutr Health Aging 2012;16:609–15. 22836701 10.1007/s12603-012-0053-9 PMC6485447

[7] Ridgway CL, Ong KK, Tammelin T, Sharp SJ, Ekelund U, Jarvelin MR Birth size, infant weight gain, and motor development influence adult physical performance. Med Sci Sports Exerc 2009;41:1212–21. 19461546 10.1249/MSS.0b013e31819794ab

[8] Rogers M, Fay TB, Whitfield MF, Tomlinson J, Grunau RE Aerobic capacity, strength, flexibility, and activity level in unimpaired extremely low birth weight (<or=800 g) survivors at 17 years of age compared with term-born control subjects. Pediatrics 2005;116:e58–65. 15997047 10.1542/peds.2004-1603

[9] Boreham CA, Murray L, Dedman D, Davey SG, Savage JM, Strain JJ Birthweight and aerobic fitness in adolescents: the Northern Ireland Young Hearts Project. Public Health 2001;115:373–9. 11781846 10.1038/sj/ph/1900800

[10] Andersen LG, Angquist L, Gamborg M, Byberg L, Bengtsson C, Canoy D, Eriksson JG, Eriksson M, Jarvelin MR, Lissner L, Birth weight in relation to leisure time physical activity in adolescence and adulthood: meta-analysis of results from 13 Nordic cohorts. PLoS ONE 2009;4:e8192. 20016780 10.1371/journal.pone.0008192 PMC2790716

[11] Vickers MH, Breier BH, McCarthy D, Gluckman PD Sedentary behavior during postnatal life is determined by the prenatal environment and exacerbated by postnatal hypercaloric nutrition. Am J Physiol Regul Integr Comp Physiol 2003;285:R271–3. 12794001 10.1152/ajpregu.00051.2003

[12] Bellinger L, Sculley DV, Langley-Evans SC Exposure to undernutrition in fetal life determines fat distribution, locomotor activity and food intake in ageing rats. Int J Obes (Lond) 2006;30:729–38. 16404403 10.1038/sj.ijo.0803205 PMC1865484

[13] Ridgway CL, Brage S, Sharp SJ, Corder K, Westgate KL, van Sluijs EM, Goodyer IM, Hallal PC, Anderssen SA, Sardinha LB, Does birth weight influence physical activity in youth? A combined analysis of four studies using objectively measured physical activity. PLoS ONE 2011;6:e16125. 21264270 10.1371/journal.pone.0016125 PMC3020226

[14] Rugholm S, Baker JL, Olsen LW, Schack-Nielsen L, Bua J, Sorensen TI Stability of the association between birth weight and childhood overweight during the development of the obesity epidemic. Obes Res 2005;13:2187–94. 16421354 10.1038/oby.2005.271

[15] Rogers IS, Ness AR, Steer CD, Wells JC, Emmett PM, Reilly JR, Tobias J, Smith GD Associations of size at birth and dual-energy X-ray absorptiometry measures of lean and fat mass at 9 to 10 y of age. Am J Clin Nutr 2006;84:739–47. 17023699 10.1093/ajcn/84.4.739

[16] Rogers I The influence of birthweight and intrauterine environment on adiposity and fat distribution in later life. Int J Obes Relat Metab Disord 2003;27:755–77. 12821960 10.1038/sj.ijo.0802316

[17] Elia M, Betts P, Jackson DM, Mulligan J Fetal programming of body dimensions and percentage body fat measured in prepubertal children with a 4-component model of body composition, dual-energy X-ray absorptiometry, deuterium dilution, densitometry, and skinfold thicknesses. Am J Clin Nutr 2007;86:618–24. 17823425 10.1093/ajcn/86.3.618

[18] Dolan MS, Sorkin JD, Hoffman DJ Birth weight is inversely associated with central adipose tissue in healthy children and adolescents. Obesity (Silver Spring) 2007;15:1600–8. 17557998 10.1038/oby.2007.189

[19] Jaiswal M, Crume T, Vehik K, Scherzinger A, Stamm E, Hamman RF, Dabelea D Is low birth weight associated with adiposity in contemporary U.S. youth? The Exploring Perinatal Outcomes among Children (EPOCH) Study. J Dev Orig Health Dis 2012;3:166–72. 23050071 10.1017/s2040174412000165 PMC3464921

[20] Kim TN, Park MS, Kim YJ, Lee EJ, Kim MK, Kim JM, Ko KS, Rhee BD, Won JC Association of low muscle mass and combined low muscle mass and visceral obesity with low cardiorespiratory fitness. PLoS ONE 2014;9:e100118. 24937121 10.1371/journal.pone.0100118 PMC4061126

[21] Ekelund U, Luan J, Sherar LB, Esliger DW, Griew P, Cooper A Moderate to vigorous physical activity and sedentary time and cardiometabolic risk factors in children and adolescents. JAMA 2012;307:704–12. Erratum in: JAMA 2012;307:1915. 22337681 10.1001/jama.2012.156 PMC3793121

[22] Ekelund U, Brage S, Besson H, Sharp S, Wareham NJ Time spent being sedentary and weight gain in healthy adults: reverse or bidirectional causality? Am J Clin Nutr 2008;88:612–7. 18779275 10.1093/ajcn/88.3.612

[23] Herrmann SD, Angadi SS Children's physical activity and sedentary time and cardiometabolic risk factors. Clin J Sport Med 2013;23:408–9. 23989385 10.1097/01.jsm.0000433154.58936.a8

[24] Hjorth MF, Chaput JP, Ritz C, Dalskov SM, Andersen R, Astrup A, Tetens I, Michaelsen KF, Sjodin A Fatness predicts decreased physical activity and increased sedentary time, but not vice versa: support from a longitudinal study in 8- to 11-year-old children. Int J Obes (Lond) 2014;38:959–65. 24304596 10.1038/ijo.2013.229

[25] Chinapaw MJ, Proper KI, Brug J, van Mechelen W, Singh AS Relationship between young peoples’ sedentary behaviour and biomedical health indicators: a systematic review of prospective studies. Obes Rev 2011;12:e621–32. 21438990 10.1111/j.1467-789X.2011.00865.x

[26] Verloigne M, Van Lippevelde W, Maes L, Yildirim M, Chinapaw M, Manios Y, Androutsos O, Kovacs E, Bringolf-Isler B, Brug J, Levels of physical activity and sedentary time among 10- to 12-year-old boys and girls across 5 European countries using accelerometers: an observational study within the ENERGY-project. Int J Behav Nutr Phys Act 2012;9:34. 22462550 10.1186/1479-5868-9-34 PMC3359200

[27] Spittaels H, van Cauwenberghe E, Verbestel V, De Meester F, Van Dyck D, Verloigne M, Haerens L, Deforche B, Cardon G, De Bourdeaudhuij I Objectively measured sedentary time and physical activity time across the lifespan: a cross-sectional study in four age groups. Int J Behav Nutr Phys Act 2012;9:149. 23249449 10.1186/1479-5868-9-149 PMC3542099

[28] Tremblay MS, LeBlanc AG, Kho ME, Saunders TJ, Larouche R, Colley RC, Goldfield G, Gorber SC Systematic review of sedentary behaviour and health indicators in school-aged children and youth. Int J Behav Nutr Phys Act 2011;8:98. 21936895 10.1186/1479-5868-8-98 PMC3186735

[29] Proper KI, Singh AS, van Mechelen W, Chinapaw MJ Sedentary behaviors and health outcomes among adults: a systematic review of prospective studies. Am J Prev Med 2011;40:174–82. 21238866 10.1016/j.amepre.2010.10.015

[30] Sherar LB, Griew P, Esliger DW, Cooper AR, Ekelund U, Judge K, Riddoch C International children's accelerometry database (ICAD): design and methods. BMC Public Health 2011;11:485. 21693008 10.1186/1471-2458-11-485 PMC3146860

[31] Riddoch C, Edwards D, Page A, Froberg K, Anderssen SA, Wedderkopp N, Brage S, Cooper AR, Sardinha LB, Harro M The European Youth Heart Study − cardiovascular disease risk factors in children: rationale, aims, study design, and validation of methods. J Phys Act Health 2005;2:115–29.

[32] Golding J, Pembrey M, Jones R ALSPAC–the Avon Longitudinal Study of Parents and Children. I. Study methodology. Paediatr Perinat Epidemiol 2001;15:74–87. 11237119 10.1046/j.1365-3016.2001.00325.x

[33] Brage S, Wedderkopp N, Ekelund U, Franks PW, Wareham NJ, Andersen LB, Froberg K Features of the metabolic syndrome are associated with objectively measured physical activity and fitness in Danish children: the European Youth Heart Study (EYHS). Diabetes Care 2004;27:2141–8. 15333475 10.2337/diacare.27.9.2141

[34] Victora CG, Hallal PC, Araujo CL, Menezes AM, Wells JC, Barros FC Cohort profile: the 1993 Pelotas (Brazil) birth cohort study. Int J Epidemiol 2008;37:704–9. 17846051 10.1093/ije/dym177

[35] Reichert FF, Menezes AM, Kingdom Wells JC, Ekelund E, Rodrigues FM, Hallal PC A methodological model for collecting high-quality data on physical activity in developing settings-the experience of the 1993 Pelotas (Brazil) Birth Cohort study. J Phys Act Health 2009;6:360–6. 19564666 10.1123/jpah.6.3.360

[36] Sardinha LB, Andersen LB, Anderssen SA, Quiterio AL, Ornelas R, Froberg K, Riddoch CJ, Ekelund U Objectively measured time spent sedentary is associated with insulin resistance independent of overall and central body fat in 9- to 10-year-old Portuguese children. Diabetes Care 2008;31:569–75. 18070991 10.2337/dc07-1286

[37] Steele RM, van Sluijs EM, Cassidy A, Griffin SJ, Ekelund U Targeting sedentary time or moderate- and vigorous-intensity activity: independent relations with adiposity in a population-based sample of 10-y-old British children. Am J Clin Nutr 2009;90:1185–92. 19776141 10.3945/ajcn.2009.28153

[38] van Sluijs EM, Skidmore PM, Mwanza K, Jones AP, Callaghan AM, Ekelund U, Harrison F, Harvey I, Panter J, Wareham NJ, Physical activity and dietary behaviour in a population-based sample of British 10-year old children: the SPEEDY study (Sport, Physical activity and Eating behaviour: environmental Determinants in Young people). BMC Public Health 2008;8:388. 19014571 10.1186/1471-2458-8-388 PMC2605463

[39] Zahner L, Puder JJ, Roth R, Schmid M, Guldimann R, Puhse U, Knopfli M, Braun-Fahrlander C, Marti B, Kriemler S A school-based physical activity program to improve health and fitness in children aged 6-13 years (“Kinder-Sportstudie KISS”): study design of a randomized controlled trial. BMC Public Health 2006;6:147. 16756652 10.1186/1471-2458-6-147 PMC1513202

[40] Ommundsen Y, Klasson-Heggebo L, Anderssen SA Psycho-social and environmental correlates of location-specific physical activity among 9- and 15- year-old Norwegian boys and girls: the European Youth Heart Study. Int J Behav Nutr Phys Act 2006;3:32. 16999865 10.1186/1479-5868-3-32 PMC1592114

[41] Troiano RP, Berrigan D, Dodd KW, Masse LC, Tilert T, McDowell M Physical activity in the United States measured by accelerometer. Med Sci Sports Exerc 2008;40:181–8. 18091006 10.1249/mss.0b013e31815a51b3

[42] Chinapaw MJ, de Niet M, Verloigne M, De Bourdeaudhuij I, Brug J, Altenburg TM From sedentary time to sedentary patterns: accelerometer data reduction decisions in youth. PLoS ONE 2014;9:e111205. 25369021 10.1371/journal.pone.0111205 PMC4219709

[43] Fischer C, Yildirim M, Salmon J, Chinapaw MJ Comparing different accelerometer cut-points for sedentary time in children. Pediatr Exerc Sci 2012;24:220–8. 22728414 10.1123/pes.24.2.220

[44] Treuth MS, Schmitz K, Catellier DJ, McMurray RG, Murray DM, Almeida MJ, Going S, Norman JE, Pate R Defining accelerometer thresholds for activity intensities in adolescent girls. Med Sci Sports Exerc 2004;36:1259–66. 15235335 PMC2423321

[45] Cole TJ, Bellizzi MC, Flegal KM, Dietz WH Establishing a standard definition for child overweight and obesity worldwide: international survey. BMJ 2000;320:1240–3. 10797032 10.1136/bmj.320.7244.1240 PMC27365

[46] World Health Organization (WHO). STEPS manual: STEPwise approach to chronic disease risk factor surveillance (STEPS). Version current 2008 Dec 12 [cited 2014 Jan 20]. Available from: http://www.who.int/chp/steps/Part3_Section3.pdf.

[47] Adegboye AR, Heitmann B Accuracy and correlates of maternal recall of birthweight and gestational age. BJOG 2008;115:886–93. 18485168 10.1111/j.1471-0528.2008.01717.x PMC2438372

[48] Preacher KJ, Hayes AF Asymptotic and resampling strategies for assessing and comparing indirect effects in multiple mediator models. Behav Res Methods 2008;40:879–91. 18697684 10.3758/brm.40.3.879

[49] Hamilton MT, Healy GN, Dunstan DW, Zderic TW, Owen N Too little exercise and too much sitting: inactivity physiology and the need for new recommendations on sedentary behavior. Curr Cardiovasc Risk Rep 2008;2:292–8. 22905272 10.1007/s12170-008-0054-8 PMC3419586

[50] Mattocks C, Deere K, Leary S, Ness A, Tilling K, Blair SN, Riddoch C Early life determinants of physical activity in 11 to 12 year olds: cohort study. Br J Sports Med 2008;42:721–4. 18780798

[51] Campbell CP, Barnett AT, Boyne MS, Soares-Wynter S, Osmond C, Fraser RA, Badaloo AV, Taylor-Bryan C, Forrester TE Predictors of physical activity energy expenditure in Afro-Caribbean children. Eur J Clin Nutr 2010;64:1093–100. 20717127 10.1038/ejcn.2010.128

[52] Hallal PC, Dumith SC, Ekelund U, Reichert FF, Menezes AM, Victora CG, Wells JC Infancy and childhood growth and physical activity in adolescence: prospective birth cohort study from Brazil. Int J Behav Nutr Phys Act 2012;9:82. 22747581 10.1186/1479-5868-9-82 PMC3458988

[53] Kehoe SH, Krishnaveni GV, Veena SR, Hill JC, Osmond C, Kiran, Coakley P, Karat SC, Fall CH Birth size and physical activity in a cohort of Indian children aged 6-10 years. J Dev Orig Health Dis 2012;3:245–52. 24098836 10.1017/S2040174412000189 PMC3790308

[54] Kim SY, Yun J Determining daily physical activity levels of youth with developmental disabilities: days of monitoring required? Adapt Phys Activ Q 2009;26:220–35. 19799095 10.1123/apaq.26.3.220

[55] Steele RM, van Sluijs EM, Sharp SJ, Landsbaugh JR, Ekelund U, Griffin SJ An investigation of patterns of children's sedentary and vigorous physical activity throughout the week. Int J Behav Nutr Phys Act 2010;7:88. 21143901 10.1186/1479-5868-7-88 PMC3018449

[56] Lee PH Association between adolescents’ physical activity and sedentary behaviors with change in BMI and risk of type 2 diabetes. PLoS ONE 2014;9:e110732. 25340773 10.1371/journal.pone.0110732 PMC4207744

[57] Metcalf BS, Hosking J, Jeffery AN, Voss LD, Henley W, Wilkin TJ Fatness leads to inactivity, but inactivity does not lead to fatness: a longitudinal study in children (EarlyBird 45). Arch Dis Child 2011;96:942–7. 20573741 10.1136/adc.2009.175927

[58] Richmond RC, Davey SG, Ness AR, den Hoed M, McMahon G, Timpson NJ Assessing causality in the association between child adiposity and physical activity levels: a Mendelian randomization analysis. PLoS Med 2014;11:e1001618. 24642734 10.1371/journal.pmed.1001618 PMC3958348

[59] Lucas A, Fewtrell MS, Cole TJ Fetal origins of adult disease-the hypothesis revisited. BMJ 1999;319:245–9. 10417093 10.1136/bmj.319.7204.245 PMC1116334

[60] Catalano PM, Ehrenberg HM The short- and long-term implications of maternal obesity on the mother and her offspring. BJOG 2006;113:1126–33. 16827826 10.1111/j.1471-0528.2006.00989.x

[61] Freeman DJ Effects of maternal obesity on fetal growth and body composition: implications for programming and future health. Semin Fetal Neonatal Med 2010;15:113–8. 19853544 10.1016/j.siny.2009.09.001

[62] Druet C, Stettler N, Sharp S, Simmons RK, Cooper C, Smith GD, Ekelund U, Levy-Marchal C, Jarvelin MR, Kuh D, Prediction of childhood obesity by infancy weight gain: an individual-level meta-analysis. Paediatr Perinat Epidemiol 2012;26:19–26. 22150704 10.1111/j.1365-3016.2011.01213.x

[63] Trost SG, Loprinzi PD, Moore R, Pfeiffer KA Comparison of accelerometer cut points for predicting activity intensity in youth. Med Sci Sports Exerc 2011;43:1360–8. 21131873 10.1249/MSS.0b013e318206476e

[64] Hildebrand M, Van Hees VT, Hansen BH, Ekelund U Age group comparability of raw accelerometer output from wrist- and hip-worn monitors. Med Sci Sports Exerc 2014;46:1816–24. 24887173 10.1249/MSS.0000000000000289

[65] Huxley R, Mendis S, Zheleznyakov E, Reddy S, Chan J Body mass index, waist circumference and waist:hip ratio as predictors of cardiovascular risk–a review of the literature. Eur J Clin Nutr 2010;64:16–22. 19654593 10.1038/ejcn.2009.68

